# Early postoperative duplex ultrasound findings of the hepatic artery in postoperative vascular complications from paediatric liver transplantation

**DOI:** 10.1007/s40477-022-00738-4

**Published:** 2022-10-20

**Authors:** Tanapong Panpikoon, Tharintorn Treesit, Chinnarat Bua-ngam, Sasikorn Feinggumloon, Kaewpitcha Pichitpichatkul, Apichaya Sriprachyakul, Satita Aimprasittichai, Apinya Chimcherd, Chollasak Thirapattaraphan, Chatmanee Lertudomphonwanit, Pornthep Tanpowpong

**Affiliations:** 1 Department of Diagnostic and Therapeutic Radiology, Faculty of Medicine, Ramathibodi Hospital, Mahidol University, 270 Rama VI Rd. Phyathai, Ratchathewi, Bangkok, 10400 Thailand; 2 Department of Surgery, Faculty of Medicine, Ramathibodi Hospital, Mahidol University, 270 Rama VI Rd. Phyathai, Ratchathewi, Bangkok, 10400 Thailand; 3 Department of Pediatrics, Faculty of Medicine, Ramathibodi Hospital, Mahidol University, 270 Rama VI Rd. Phyathai, Ratchathewi, Bangkok, 10400 Thailand

**Keywords:** Paediatric liver transplantation, Hepatic artery stenosis, Hepatic artery thrombosis, Portal vein stenosis, Portal vein thrombosis, HV-IVC stenosis, HV-IVC thrombosis, Duplex ultrasound

## Abstract

**Purpose:**

To evaluate duplex US findings of the HA in all three postoperative vascular (HA, PV, HV and IVC) complications of paediatric LT for early detection and some helpful secondary signs to determine these vascular complications.

**Materials and methods:**

We collected data from 44 post-LT paediatric patients who underwent daily duplex US for seven consecutive days and three months after LT during January 2017–June 2020. Four duplex US parameters of the HA (extrahepatic PSV, intrahepatic PSV, RI and AT) were compared in patients with and without complications.

**Results:**

The PSV of the extrahepatic HA in patients with HA complications was higher than that in patients without complications (*P* value = 0.019). The PSV at 107.7 cm/s is the optimal cut-off parameter associated with HA complications [a sensitivity of 88.9% and a specificity of 80.0% (ROC area is 0.84)]. The intrahepatic RI was higher on the first day than on the last day and gradually decreased in patients without vascular complications (*P* value = 0.000). The intrahepatic PSV significantly decreased with time when comparing the first and last days in patients without PV and HV-IVC complications (*P* value = 0.014 and 0.038). In contrast, patients with vascular complications showed no significant decrease.

**Conclusion:**

The extrahepatic PSV relates to HA complications after paediatric LT but not PV and HV-IVC complications. Non-significantly decreased intrahepatic RI and PSV from the first day to the day of complication diagnosis may correlate with the occurrence of vascular complications.

## Key Points

**Table:** 

The PSV of the extrahepatic HA in patients with HA complications was higher than that in patients without complications (*P* value = 0.019).
The PSV of the extrahepatic HA at 107.7 cm/sec is the optimal cut-off parameter associated with HA complications [a sensitivity of 88.9% and a specificity of 80.0% (ROC area is 0.84)].
The intrahepatic RI was higher on the first day than on the last day and gradually decreased in patients without vascular complications (*P* value = 0.000).
The intrahepatic PSV significantly decreased with time when comparing the first and last days in patients without PV and HV-IVC complications (*P* value = 0.014 and 0.038).

## Introduction

In the past decade, liver transplantation (LT) has gained acceptance as a definitive treatment for end-stage liver disease (ESLD) [1] in both paediatric and adult patients. Children account for only 7.8% of all liver transplants [2], mainly due to specific challenges, such as more complex surgeries and paediatric pre-existing liver conditions, including congenital, metabolic and oncological diseases [3, 4]. Recently, advancements in surgical techniques and equipment and pre- and post-operative management have dramatically improved liver transplantation, resulting in better graft survival rates, decreased morbidity or mortality rates, and fewer postoperative graft-related complications.

Vascular complications (VCs) are the most severe complication and a common cause of graft failure after LT [5]. Early detection and intervention of postoperative vascular complications are crucial for improving graft and patient survival [6]. Fortunately, duplex ultrasonography (US) has proven effective in detecting and predicting multiple graft-related complications, such as early vascular thrombosis and acute graft rejection, even before patients develop clinical signs [7–10]. It is widely accessible, cost-effective, and radiation-free and can be performed at the bedside. Thus, duplex US has played an essential role in the postoperative surveillance of LT in children and is the initial posttransplantation imaging modality for the detection and follow-up of early and delayed VCs.

Knowledge of the transplanted vascular anatomy and the physiologic graft haemodynamics after graft perfusion are essential for the early identification of VCs by duplex US. The three main transplanted vascular systems that need to be well-known are the hepatic arterial (HA) system, portal venous (PV) system and hepatic vein-inferior vena cava (HV-IVC) system. For example, if any complications are occurring in one of these vascular systems, that is, either hepatic artery thrombosis or stenosis, the change in duplex US findings [e.g., waveforms, peak systolic velocity (PSV), resistance index (RI), or acceleration time (AT)] of the HA system will be detected, leading to the diagnosis of complications in that specific vessel. However, when a problem occurs in one vascular system, it will theoretically impact the haemodynamics of that system and the whole haemodynamics of the transplanted liver. For instance, when the PV is thrombosed, hepatic arterial compensation is seen as increased arterial blood flow or multiple hepatic collaterals/cavernous transformation (hepatic arterial buffer response), which maintains enough blood supply to the transplanted liver. In addition, a reduction in the hepatic artery RI is also found to correlate with PV thrombosis [11]. In another case, when HV-IVC thrombosis or stenosis occurs, leading to hepatic venous outflow tract obstruction and liver congestion, hepatic arterial blood flow is supposed to decrease due to the rising intrahepatic venous pressure. Thus, the duplex US parameters measured in the HA are thought to be changed in the case of PV or HV-IVC thrombosis and stenosis according to the effect of one affected vascular system on the entire haemodynamics of the transplanted liver and might be helpful secondary signs to determine these vascular abnormalities.

Hence, we designed this study to evaluate whether abnormal duplex US findings of the HA (extrahepatic hepatic artery PSV, intraparenchymal hepatic artery PSV, RI and AT) can be used for the early diagnosis of postoperative vascular complications.

## Materials and methods

Study design: Retrospective cohort study.

The Institutional Review Boards approved this study, and the requirement for informed consent was waived.

All data were collected retrospectively from electronic medical records (EMRs) and the picture achieving and communication system (PACS), including all paediatric patients who underwent liver transplantation from January 2017 to June 2020. Both the whole liver transplantation technique and the split living-donor liver transplantation technique performed in these patients were included. Patients who had incomplete duplex US data or were lost to follow-up were excluded from this study.

### Inclusion criteria

1. All paediatric patients who underwent liver transplantation from January 2017 to June 2020.
2. Complete information of interval follow-up by duplex US in the first 7 days after liver transplantation and the next three months.
3. Regular follow-up performed for at least 3 months after the operation.

### Exclusion criteria

1. Paediatric liver-transplant patients with no official report of postoperative duplex US findings provided by radiologists.
2. Lack of regular follow-up.

The collected data included in this study consisted of demographic data of the patients (recipients), such as age at the date of transplantation, sex, height, weight, blood group, underlying disease(s), indication for liver transplantation, last MELD score, date of liver transplantation, post-transplant complications, and official report of duplex US measurements and spectral Doppler characteristics, such as flow velocity, RI, or AT. The only vascular-related complication included in this study was HA, PV or HV-IVC stenosis or thrombosis. All patients were examined via an ultrasonic diagnostic device (Aplio™ 500, Canon Medical Systems Corporation, Japan) with a 3.5 MHz convex transducer (PVT-375BT) on the first consecutive seven days after the operation and on the date of the 3-month follow-up. Radiology residents initially performed all examinations. Repeated duplex ultrasound examination by a consultant radiologist and further CTA or angiography of the transplanted liver was performed to provide more detailed information or confirm the diagnosis if abnormal US parameters were detected.

The scanning protocol starts with greyscale US for the liver parenchyma and each vascular structure, including anastomosis of HA, PV and HV–IVC, followed by colour and spectral waveform Doppler US. The Doppler signal was sampled by placing the sample gate at the preanastomotic and anastomotic sites. Vascular anastomotic stenosis was suspicious if a markedly decreased luminal diameter in the B-mode examination or aliasing in the colour flow image were detected. The preanastomotic site was 1–2 cm proximal to the anastomosis.

The criteria for diagnosing HA, PV and HV-IVC thrombosis were intraluminal echogenicity or no detectable colour flow in the vascular lumen. HA stenosis was diagnosed when the anastomotic/preanastomotic PSV ratio was greater than 2.0. PV and HV-IVC stenoses were diagnosed when the anastomotic/preanastomotic velocity ratio was greater than 3.0.

Normal duplex US findings of the HA, PV and HV-IVC in the first seven consecutive days and during the follow-up at 3-month intervals after liver transplantation were considered to indicate no complications. If any abnormal duplex US parameter of the HA, PV or HV-IVC was detected and confirmation with CTA or angiography of the transplanted liver was definite, vascular complications were considered.

STATA^®^ statistical software version 18.0 was used to analyse the data. Categorical variables, sex and blood group were evaluated by chi-squared tests and are presented as frequencies and percentages. Other demographic data, which were continuous data, are represented as the mean. The one-sample Kolmogorov‒Smirnov test, Mann‒Whitney test and Wilcoxon signed ranks test were used to compare data. The results are expressed as the median (min–max). A p value less than 0.05 was considered statistically significant.

## Results

A total of 44 paediatric patients (19 boys and 25 girls) were included in this study. Twenty-seven of 44 patients had normal graft status after follow-up with a paediatric doctor over three months, with normal laboratory and duplex US examination. The age range was 3–207 months, with a mean age of 41 months. The patients' body weights ranged from 5.2 to 55.0 kg (mean, 13.8 kg), with BMIs ranging from 12.3 to 22.6 (mean, 16.1). Other demographic data are shown in Table 1.

**Table 1:** Demographic data

Demographic data	Number (percentage)	Range	Mean
Gender			
Male	19, 43.2%		
Female	25, 56.8%		
Age (months)		3–207	41
Blood group			
A	7, 15.9%		
B	17, 38.6%		
AB	2, 4.5%		
O	18, 40.9%		
Height (cm)		62–158	86.9
Weight (kg)		5.2–55.0	13.8
BMI		12.3–22.6	16.1
Last MELD		2–29	17.9
Indication of liver transplantation			
Acute liver failure	1		
Acute top chronic liver failure	31		
Cirrhosis	29		
Liver cancer	2		
Primary liver disease			
Cholestatic disease	34		
Non-viral hepatitis	1		
Metabolic liver disease	1		
Another acute liver failure	2		
Liver mass	1		
Previous operation			
Hepatic resection	26		
Hepatobiliary bypass	1		
PTBD	3		

HA complications were found in 9 of 44 patients (20.5%). One of them was found to have both HA and HV-IVC thromboses. Six patients had HA stenosis, and two had HA thrombosis.

Five of 44 patients (11.4%) had PV complications. Two of them were found to have both PV and HV-IVC complications. There were two patients with PV stenosis alone and one with PV thrombosis.

Six of 44 patients (13.6%) had HV-IVC complications. Three of them had HV-IVC stenosis. The other three patients with combined HA or PV complications were described earlier. All patients with vascular complications were initially diagnosed with duplex US and confirmed by CT.

Our study divided patients into 6 study groups: patients with HA complications, patients without HA complications, patients with PV complications, patients without PV complications, patients with HV-IVC complications, and patients without HV-IVC complications. Four duplex US parameters of HA evaluated in this study were extrahepatic PSV, intrahepatic (intraparenchymal) PSV, intrahepatic RI and intrahepatic AT. The mean of each parameter from the first to the last day was used for analysis in the patients without complications and from the first day to the day of complication detection within the first seven consecutive days after liver transplantation in patients with vascular complications.

The extrahepatic PSV showed a statistically significant difference between patients with and without HA complications (*P* value = 0.005). The intrahepatic PSV, RI and AT showed no statistically significant difference between the two groups (*P* value = 0.631, 0.771 and 0.134, respectively).

In the patients with and without PV complications, all four duplex US parameters of the HA showed no statistically significant difference between the two groups. Similarly, in the patients with and without HV-IVC complication groups, all duplex US parameters of the HA also showed no statistically significant difference between the two groups.

The duplex US parameters of the HA in patients with and without vascular complications are shown in Table 2.

**Table 2:** Comparison of duplex US parameters of the hepatic artery in patients with and without vascular complications

Duplex ultrasound parameters	Hepatic artery complication			Portal vein complication			HV-IVC complication		
	Yes (*N* = 9)	No (*N* = 35)	*P*-value	Yes (*N* = 5)	No (*N* = 39)	*P*-value	Yes (*N* = 6)	No (*N* = 38)	*P*-value
Extra-hepatic hepatic artery (pre-anastomotic site)									
1. PSV	133.5 (25.6–295.2)	91.7 (51.5–161.0)	0.005*	81.5 (53.1–149.7)	96.3 (25.6–295.2)	0.448	100.0 (78.0–154.4)	95.3 (25.6–295.2)	0.538
Intra-hepatic hepatic artery (intraparenchymal hepatic artery)									
2. PSV	40.4 (14.0–132.3)	43.3 (23.6–81.9)	0.631	40.4 (26.9–71.8)	43.3 (14.0–132.3)	0.956	35.2 (23.6–53.3)	45.3 (14.0–132.3)	0.060
3. RI	0.78 (0.60–0.90)	0.76 (0.60–0.90)	0.771	0.75 (0.60–0.90)	0.76 (0.60–0.90)	0.343	0.73 (0.60–0.90)	0.76 (0.60–0.90)	0.958
4. AT	0.046 (0.024–0.114)	0.038 (0.025–0.083)	0.134	0.033 (0.025–0.050)	0.039 (0.024–0.114)	0.630	0.040 (0.033–0.061)	0.039 (0.024–0.114)	0.273

Except where indicated otherwise, data are reported as median (Min–Max)

*Statistically significant at *P* < 0.05

The extrahepatic PSV in patients with HA complications was higher than that in patients without complications [133.5 (25.6–295.2) vs. 91.7 (51.5–161.0), OR (95% CI) = 1.030 (1.005–1.056), P value = 0.019]. The receiver operating characteristic (ROC) curve was constructed to determine the extrahepatic PSV that maximizes the summation of sensitivity and specificity to detect HA complications. The extrahepatic PSV at 107.7 cm/s is the optimal cut-off parameter associated with HA complications, with a sensitivity of 88.9% and a specificity of 80.0% [ROC area is 0.84, SE (95% CI) 0.07 (0.71,0.99)].

Differences between the mean of each parameter at the first and the day of complication detection within the first seven consecutive days after liver transplantation were also analysed in each group. The PSV of the extrahepatic HA showed no statistically significant difference between the patients with complications and those without complications.

The intrahepatic PSV significantly decreased with time when the comparison was performed between the first- and last-day data in patients without PV complications [49.3 (1.8–194.3) vs. 36.3 (0.9–179.8), *P* value = 0.014]. A similar result was found in patients without HV-IVC complications [51.0 (1.8–194.3) vs. 38.8 (0.9–179.8), *P* value = 0.038].

The intrahepatic RI measured on the first day was high, with a gradual decrease with time compared to the last day data in patients without vascular complications. The intrahepatic RI measured on the first and last days showed a statistically significant difference in patients without HA complications [0.80 (0.60–1.00) vs. 0.70 (0.50–0.90), *P* value = 0.000], in patients without PV complications [0.80 (0.40–1.00) vs. 0.70 (0.40–0.90), *P* value = 0.000] and in patients without HV-IVC complications [0.80 (0.40–1.00) vs. 0.70 (0.40–0.90), *P* value = 0.000]. This was contrary to the results from patients with vascular complications, in whom the PSV and RI of the intraparenchymal HA showed no statistically significant difference between the first and last days.

The US parameters measured on the first and last days during the study period in our six patient groups are shown in Table 3.

**Table 3:** The duplex US parameters measured on the first and last days in the six study groups

Duplex ultrasound parameters	Hepatic artery complication						Portal vein complication						HV-IVC complication					
	Yes (*N* = 9)			No (*N* = 35)			Yes (*N* = 5)			No (*N* = 39)			Yes (*N* = 6)			No (*N* = 38)		
	1st day	Last day	*P*-value	1st day	Last day	*P*-value	1st day	Last day	*P*-value	1st day	Last day	*P*-value	1st day	Last day	*P*-value	1st day	Last day	*P*-value
Extra-hepatic hepatic artery (Pre-anastomotic site)																		
1. PSV	106.8 (32.2–295.2)	208.7 (19.0–409.7)	0.069	85.2 (2.7–262.3)	79.6 (15.5–417.0)	0.456	69.2 (53.1–154.7)	81.5 (53.1–230.0)	0.655	89.9 (2.7–295.2)	99.8 (15.5–417.0)	0.134	90.7 (42.3–177.7)	127.7 (47.6–230.0)	0.068	86.1 (2.7–295.2)	87.0 (15.5–417.0)	0.253
Intra-hepatic hepatic artery (intraparenchymal hepatic artery)																		
2. PSV	43.4 (17.5–185.8)	34.9 (5.5–78.8)	0.058	52.5 (1.8–194.3)	41.3 (0.9–179.8)	0.096	40.4 (22.1–71.8)	66.5 (25.1–71.8)	0.180	49.3 (1.8–194.3)	36.3 (0.9–179.8)	0.014*	38.0 (23.6–89.3)	32.0 (5.5–68.1)	0.273	51.0 (1.8–194.3)	38.8 (0.9–179.8)	0.038*
3. RI	0.80 (0.40–0.90)	0.80 (0.40–0.90)	0.167	0.80 (0.60–1.00)	0.70 (0.50–0.90)	0.000*	0.80 (0.60–0.90)	0.70 (0.60–0.90)	0.157	0.80 (0.40–1.00)	0.70 (0.40–0.90)	0.000*	0.80 (0.60–0.90)	0.65 (0.40–0.90)	0.465	0.80 (0.40–1.00)	0.70 (0.40–0.90)	0.000*
4. AT	0.039 (0.017–0.050)	0.044 (0.022–0.189)	0.080	0.039 (0.022–0.067)	0.039 (0.017–0.074)	0.770	0.033 (0.022–0.050)	0.033 (0.028–0.056)	0.180	0.039 (0.017–0.067)	0.039 (0.017–0.189)	0.640	0.040 (0.022–0.044)	0.039 (0.022–0.117)	0.109	0.039 (0.017–0.067)	0.039 (0.017–0.189)	0.550

Except where indicated otherwise, data are reported as median (Min–Max)

## Discussion

Currently, vascular complications after paediatric liver transplantation still occur despite improvements in medical and surgical techniques. Duplex US has played an essential role in the postoperative surveillance of LT in children and is the initial posttransplantation imaging modality for detecting and following vascular complications. Thus, we designed this study to evaluate whether the duplex US parameters of the HA can be used for early detection in all three postoperative vascular complications (the HA itself and PV and HV-IVC thrombosis or stenosis).

The duplex US parameters of the HA were chosen for evaluation in this study because the characteristic waveform signature is the easiest to understand and evaluate among all three hepatic vasculatures. The HA waveform is ordinarily pulsatile with low resistance and shows antegrade flow throughout the entire cardiac cycle, always being displayed above the baseline. These make HA duplex US parameters much easier and more reliable than those of the PV and HV, which can be simply changed in waveform pattern and velocity due to external factors such as a patients' position and respiratory cycle.

In this study, the extrahepatic PSV in patients with HA complications was higher than that in patients without complications (*P* value = 0.019). The PSV at 107.7 cm/s is the optimal cut-off parameter associated with HA complications (a sensitivity of 88.9%, a specificity of 80.0%, and a ROC area of 0.84). In comparison, previous studies [12–14] reported an extrahepatic PSV of 50–200 cm/s in normal graft status within the first year after liver transplantation, and a PSV > 200 cm/s was associated with HA complications. This could be due to the difference in the study population. Primarily, we studied the first week after transplantation, and the mean value measured from the first day to the last day after transplantation was used for statistical analysis.

The intrahepatic RI was higher on the first day than on the last day and gradually decreased in patients without HA, PV or HV-IVC complications. These results are explained by resolving postoperative factors such as postoperative oedema of the liver parenchyma (reperfusion oedema), soft tissue oedema around the anastomotic site and vasospasm. This observation is congruent with previous studies [14–16]. Our study reports that the intrahepatic RI measured on the first and last days showed a statistically significant difference in patients without HA, PV or HV-IVC complications (*P* value = 0.000 in all groups).

Theoretically, when a problem occurs in one vascular system after LT, it will impact not only the haemodynamics of that system but also the whole haemodynamics of the transplanted liver. In 1995, Platt JF et al. reported that a reduction in the hepatic artery RI accompanies PV thrombosis and may be a helpful secondary sign to determine this venous abnormality [11]. In 2021, Gaillard et al. reported an increased hepatic artery RI, more than 0.75, in hepatic venous outflow obstruction (HVOO) [17]. Thus, we thought that if thrombosis or stenosis occurs within the PV or HV-IVC system, abnormalities in the hepatic artery RI should be found and may help diagnose these venous problems.

**Fig. 1:**
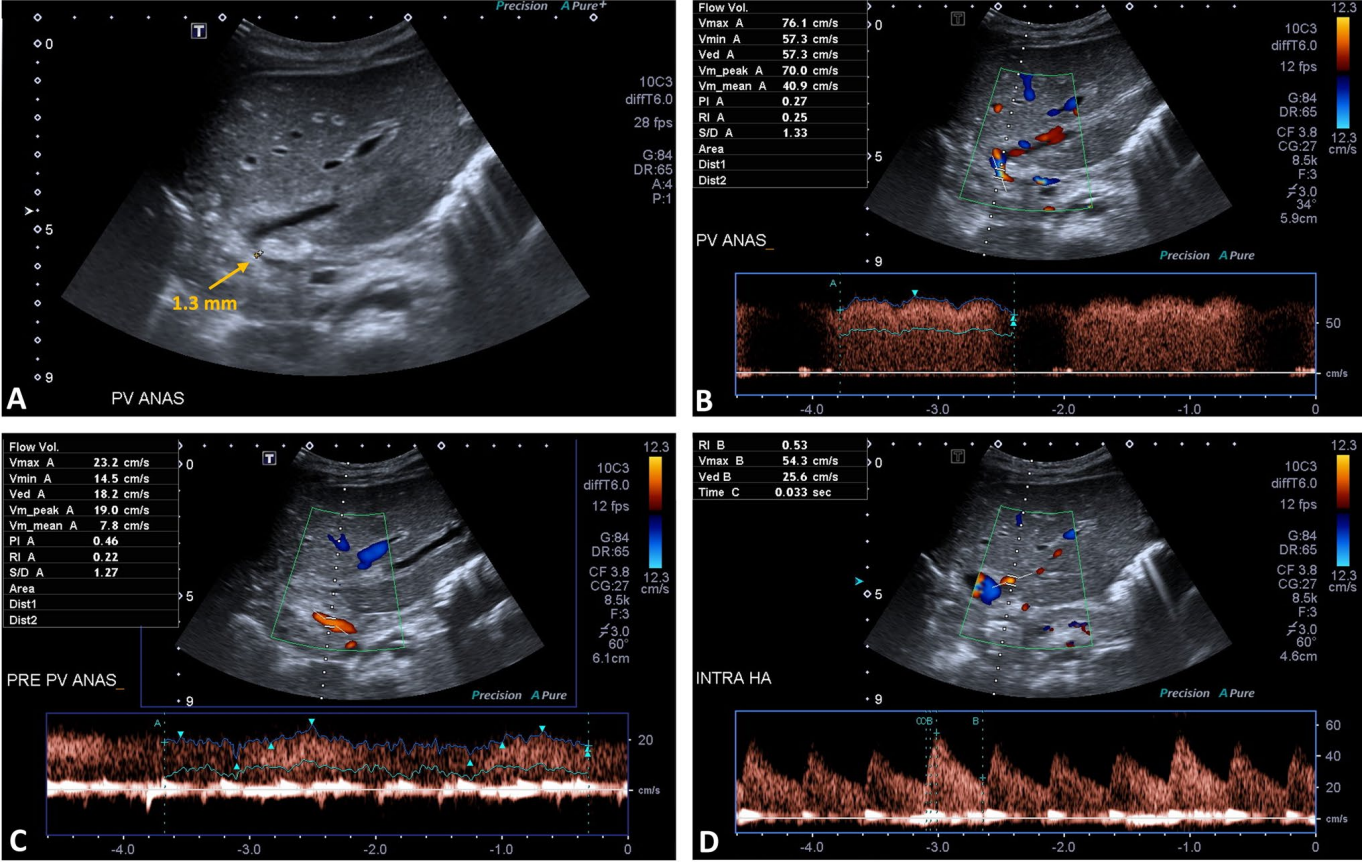
A 3-month-old female infant with post-liver-transplantation portal vein stenosis on the 5th day after surgery. **A** Grey scale US examination showing severe stenosis of the PV anastomotic site, measuring 1.3 mm in diameter. **B**, **C** Duplex US examination showing a fivefold increased mean velocity at the PV anastomotic site over that at the pre-anastomotic site, representing hemodynamically significant stenosis of the PV anastomosis. **D** Duplex US examination showing decreased intra-hepatic RI, measured as 0.53

**Fig. 2:**
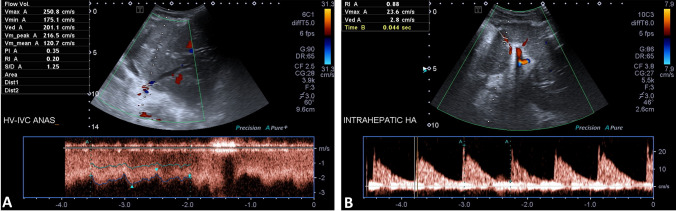
A 23-month-old female toddler with post-liver-transplantation HV-IVC anastomotic stenosis on the 1st day after surgery. **A** Duplex US examination showing a 16-fold increased mean velocity at the HV-IVC anastomotic site over that at the HV pre-anastomotic site. **B** Duplex US examination showing a significantly increased intra-hepatic RI, measured as 0.88

Our study revealed that intrahepatic RI showed no statistically significant difference among the patients with and without PV or HV-IVC complications. This finding can be due to using the mean value measured from the first day after LT until the day of vascular complication detection for analysis instead of the exact value recorded on the day of complication diagnosis. When we directly determined the precise value of intrahepatic RI on the day of PV thrombosis detection in some cases from our study, it revealed that the intrahepatic RI was decreased, correlating with the prior study result (Fig. 1). The intrahepatic RI measured on the day of HV-IVC stenosis detection also showed a significant increase (Fig. 2).

In addition, in patients without vascular complications, the intrahepatic RI and PSV were measured from the first to the seventh consecutive days after LT. However, in patients with vascular complications, most of these adverse events usually occur in the very first days after LT, especially the first and second days. This might explain why the hepatic artery RI and PSV values measured on the first day and on the day of complication detection showed no statistically significant difference. Moreover, the small sample size in our study might have contributed to the inadequate data and made us unable to divide the vascular complication groups into thrombosis and stenosis subgroups, which could be another cause of the lack of a statistically significant difference in the results.

However, the sequential change in HA parameters within the first seven days after the operation can predict vascular complications. For example, the absence of a decrease in intrahepatic RI or PSV during the first 7 days after the procedure may be related to vascular complications.

There are several limitations in our study other than those mentioned earlier. The appropriate distribution and accuracy of each duplex US parameter depend on the examiner's ultrasound experiences. A small sample size with a single centre retrospective cohort study design may affect the cut-off value calculation and parameter comparison. Further large-scale studies with prospective study designs are necessary to evaluate the efficacy and usefulness of duplex US parameters of HA in the early detection and diagnosis of the posttransplanted HA itself and PV and HV-IVC complications.

## Conclusion

The PSV of the extrahepatic HA in patients with HA complications was higher than that in patients without complications (P value = 0.019). The PSV at 107.7 cm/s was the optimal cut-off parameter associated with HA complications, with a sensitivity of 88.9% and a specificity of 80.0% (ROC area: 0.84). The intrahepatic RI measured on the first day was higher than that measured on the last day, with a gradual decrease in patients without HA, PV or HV-IVC complications (*P* value = 0.000 in all groups). The intrahepatic PSV significantly decreased with time when comparing the first and last days in patients without PV and HV-IVC complications (*P* value = 0.014 and 0.038). In contrast, patients with vascular complications showed no significant decrease. Non-significantly decreased intrahepatic RI and PSV on the day of vascular complication diagnosis compared to those on the first day of measurement in patients with vascular complication groups may correlate with vascular complications.

## Data Availability

The datasets analysed during the current study are available from the corresponding author on reasonable request.

## Abbreviations

AT: Acceleration time
BMI: Body mass index
CT: Computer tomography
EDV: End-diastolic velocity
ERCP: Endoscopic retrograde cholangiopancreatography
HA: Hepatic artery
HV–IVC: Hepatic vein–inferior vena cava
MELD: Model for End-Stage Liver Disease
MRI: Magnetic resonance imaging
N: Number
OR: Operation room
PV: Portal vein
PSV: Peak systolic velocity
RI: Resistance index
ROC: Receiver operating characteristic
SE: Standard error
US: Ultrasonography

## References

[1] Busuttil RW, Farmer DG, Yersiz H, Hiatt JR, McDiarmid SV, Goldstein LI (2005). Analysis of long-term outcomes of 3200 liver transplantations over two decades: a single-centre experience. Ann Surg.

[2] Squires RH, Ng V, Romero R, Ekong U, Hardikar W, Emre S (2014). Evaluation of the pediatric patient for liver transplantation: 2014 practice guideline by the American Association for the Study of Liver Diseases, American Society of Transplantation and the North American Society for Pediatric Gastroenterology. Hepatol Nutr Hepatol.

[3] Hadžić N, Baumann U, McKiernan P, McLin V, Nobili V (2017). Long-term challenges and perspectives of pre-adolescent liver disease. Lancet Gastroenterol Hepatol.

[4] McDiarmid SV, Anand R, Martz K, Millis MJ, Mazariegos G (2011). A multivariate analysis of pre-, peri-, and post-transplant factors affecting outcome after pediatric liver transplantation. Ann Surg.

[5] Duffy JP, Hong JC, Farmer DG, Ghobrial RM, Yersiz H, Hiatt JR (2009). Vascular complications of orthotopic liver transplantation: experience in more than 4200 patients. J Am Coll Surg.

[6] Bekker J, Ploem S, de Jong KP (2009). Early hepatic artery thrombosis after liver transplantation: a systematic review of the incidence, outcome and risk factors. Am J Transplant.

[7] Britton PD, Lomas DJ, Coulden RA, Farman P, Revell S (1992). The role of hepatic vein Doppler in diagnosing acute rejection following paediatric liver transplantation. Clin Radiol.

[8] Gu LH, Fang H, Li FH, Li P, Zhu CX, Zhu JJ (2012). Prediction of early hepatic artery thrombosis by intraoperative colour Doppler ultrasound in pediatric segmental liver transplantation. Clin Transpl.

[9] Jéquier S, Jéquier JC, Hanquinet S, Le Coultre C, Belli DC (2003). Orthotopic liver transplants in children: change in hepatic venous Doppler wave pattern as an indicator of acute rejection. Radiology.

[10] Kok T, Slooff MJ, Thijn CJ, Peeters PM, Verwer R, Bijleveld CM (1998). Routine Doppler ultrasound for the detection of clinically unsuspected vascular complications in the early postoperative phase after orthotopic liver transplantation. Transpl Int.

[11] Platt JF, Rubin JM, Ellis JH (1995). Hepatic artery resistance changes in portal vein thrombosis. Radiology.

[12] Dodd GD, Memel DS, Zajko AB, Baron RL, Santaguida LA (1994). Hepatic artery stenosis and thrombosis in transplant recipients: Doppler diagnosis with resistive index and systolic acceleration time. Radiology.

[13] Abdelaziz O, Attia H (2016). Doppler ultrasonography in living donor liver transplantation recipients: Intra- and postoperative vascular complications. World J Gastroenterol.

[14] Ahmad T, Chavhan GB, Avitzur Y, Moineddin R, Oudjhane K (2017). Doppler parameters of the hepatic artery as predictors of graft status in pediatric liver transplantation. AJR Am J Roentgenol.

[15] Jamieson LH, Arys B, Low G, Bhargava R, Kumbla S, Jaremko JL (2014). Doppler ultrasound velocities and resistive indexes immediately after pediatric liver transplantation: normal ranges and predictors of failure. AJR Am J Roentgenol.

[16] Park YS, Kim KW, Lee SJ, Lee J, Jung DH, Song GW (2011). Hepatic arterial stenosis assessed with doppler US after liver transplantation: frequent false-positive diagnoses with tardus parvus waveform and value of adding optimal peak systolic velocity cut-off. Radiology.

[17] Gaillard F, Skalina, T. Budd-Chiari syndrome. Radiopaedia.org.2008 [updated 27 Oct 2021]. https://radiopaedia.org/articles/budd-chiari-syndrome-1?lang=us.

